# Effects of capsaicin and monensin on ruminal fermentation, intake, nutrient digestibility, and ruminal dynamics of grazing bulls

**DOI:** 10.5194/aab-69-275-2026

**Published:** 2026-05-05

**Authors:** Murilo R. Santiago, Mónica Madrigal-Valverde, Ana Paula G. da Silva, Maria Leonor G. M. L. de Araújo, Gleidson Giordano P. de Carvalho, Douglas dos Santos Pina, Aureliano José V. Pires, Artur A. Menezes, Lara L. Dantas, Maria Luiza O. Chaves, Lara Maria S. Brant, José Esler de Freitas Júnior

**Affiliations:** 1 Department of Animal Science, Federal University of Bahia, Salvador, 40.170-010, Brazil; 2 Instituto Tecnológico de Costa Rica, Alajuela, 223-21001, Costa Rica; 3 Department of Animal Science, State University of Southeast Bahia, Itapetinga, 45.700-000, Brazil

## Abstract

Dietary supplementation with feed additives may influence intake, nutrient digestibility, and ruminal fermentation in grazing cattle. The objective of this study was to evaluate the effects of supplementation with capsaicin and/or monensin on intake, nutrient digestibility, energy balance, blood parameters, ruminal kinetics, ingestive behavior, and ruminal fermentation of grazing bulls. Four crossbred (Holstein × Zebu) bulls (375 ± 14 kg body weight (BW); 24 ± 1 months of age), fitted with rumen cannulas, were assigned to a 4 × 4 Latin square design and maintained on 1.2 ha of Pangola grass (*Digitaria decumbens*) pasture with supplementation at 0.3 % of BW. The bulls were allocated to the following treatments: (1) control (CON): supplementation without additives; (2) capsaicin (CAP): supplementation including 100 mg kg^−1^ of total dry matter (DM) of capsaicin (CAPCIN^®^, NutriQuest, São Paulo, Brazil); (3) monensin (MON): supplementation including 20 mg kg^−1^ of total DM of sodium monensin (Poulcox 40^®^, Huvepharma, Bulgaria); and (4) capsaicin + monensin (CAPMON): supplementation including sodium monensin (20 mg kg^−1^ DM) and capsaicin (100 mg kg^−1^ DM). Supplementation with CAP and MON increased the total tract digestibility of organic matter (OM), crude protein (CP), and neutral detergent fiber (NDF) compared with CAPMON supplementation. A tendency toward a greater ruminal DM pool was observed in bulls supplemented with CAPMON compared with those receiving CAP and MON, whereas bulls supplemented with MON showed higher passage and turnover rates of potentially digestible neutral detergent fiber (_td_NDF) than those supplemented with CAP. Bulls receiving CAPMON spent more time grazing (min d^−1^) compared with those supplemented with CAP and MON. In addition, bulls supplemented with CAP and MON exhibited higher ruminal pH and lower propionate concentration than those supplemented with CAPMON. The use of capsaicin and/or monensin at the evaluated doses may be applied in animal production.

## 1 Introduction

Energy efficiency in beef cattle production under tropical pasture-based systems is limited by seasonal variability in forage quality, ruminal fermentation dynamics, and inherent energy losses associated with microbial metabolism, particularly enteric methane production (Beauchemin et al., 2020). In cattle fed predominantly tropical forages, the higher proportion of structural carbohydrates promotes fermentative patterns characterized by increased acetate and hydrogen production, which intensifies methanogenesis and reduces the efficiency of metabolizable energy utilization (Sun et al., 2022).

In this context, the use of feed additives has been widely investigated as a strategy to modulate ruminal fermentation, improve dietary energy utilization, and enhance animal performance (Dorantes-Iturbide et al., 2022; Al Rharad et al., 2025). These additives can be classified as nutritional (vitamins and minerals); zootechnical (enzymes, prebiotics, probiotics, and ionophore antibiotics); and functional or health-promoting additives, such as organic acids, microalgae, and phytogenic compounds (essential oils, tannins, saponins, and capsaicin), which act through different mechanisms on the ruminal microbiota and host metabolism (Pimentel et al., 2022; Boukrouh et al., 2025).

Among phytogenic additives, plant-derived bioactive compounds have attracted increasing interest as alternatives or complements to ionophores. Capsaicin, a phenolic alkaloid present in peppers of the genus *Capsicum*, has been investigated as a functional additive in ruminant diets due to its pungent properties, which may influence feeding behavior, water intake, and ruminal fermentation dynamics (Rodríguez-Prado et al., 2012). However, the biological responses to capsaicin appear to be dose dependent. Studies with dairy cattle have evaluated a wide range of supplementation levels, typically from 100 to 2000 mg animal^−1^ d^−1^ depending on the form of the additive and the production system. In general, doses of around 100 to 200 mg animal^−1^ d^−1^ of protected capsaicin have been associated with improvements in milk production, feed efficiency, and metabolic status (Oh et al., 2016, 2021), whereas higher inclusion levels, such as 1200 mg animal^−1^ d^−1^, have been reported to enhance lactation performance, immune responses, and oxidative status in dairy cows (Li et al., 2023). In beef cattle, capsaicin supplementation has also been evaluated at different inclusion levels, with responses varying according to dose and dietary conditions. Westphalen et al. (2021) evaluated rumen-protected *Capsicum oleoresin* at doses ranging from 5 to 15 mg kg^−1^ of dietary dry matter and reported improvements in weight gain and feed efficiency, suggesting beneficial effects at moderate inclusion levels. Similarly, You et al. (2025) supplemented beef cattle with 250 mg animal^−1^ d^−1^ of capsaicin and observed stabilization of ruminal fermentation parameters, including increased ruminal pH. In grazing systems, Lopes et al. (2025) reported that supplementation with approximately 450 mg animal^−1^ d^−1^ of microencapsulated pepper improved nutrient utilization and ruminal fermentation responses in beef cattle. Likewise, Miranda et al. (2025) observed that the inclusion of 450 mg animal^−1^ d^−1^ of microencapsulated pepper in supplements offered at different energy levels (0.15 %, 0.30 %, and 0.45 % of body weight) influenced ruminal fermentation, particularly by reducing ruminal ammonia concentration, although no effects were detected on dry matter intake or nutrient digestibility. Together, these findings indicate that the magnitude of the response to capsaicin supplementation in beef cattle depends on the inclusion level and dietary conditions.

Ionophore antibiotics, such as monensin, are widely used to improve energy efficiency in ruminants by selectively modulating the ruminal microbiota and favoring propionate production at the expense of acetate. This fermentative shift increases energy availability to the animal and improves feed efficiency (Pimentel et al., 2022). Nevertheless, concerns regarding microbial resistance and regulatory restrictions have stimulated the investigation of alternative or combined strategies involving natural additives (Orzuna-Orzuna et al., 2022).

The association between capsaicin and monensin may represent a complementary approach, as these additives act through distinct mechanisms. While monensin exerts a direct and consistent effect on ruminal fermentation and fermentative efficiency, capsaicin may indirectly modulate the microbiota, and influence fermentative and metabolic responses. This combination may potentiate the effects on nutrient intake, digestibility, and energy utilization efficiency in grazing cattle.

Therefore, it was hypothesized that supplementation with capsaicin, alone or in combination with monensin, could modulate ruminal fermentation and improve nutrient intake and digestibility in grazing bulls. Accordingly, the objective of this study was to evaluate the effects of supplementation with capsaicin and/or monensin on intake, nutrient digestibility, energy balance, blood parameters, ruminal kinetics, ingestive behavior, and ruminal fermentation in grazing bulls.

## 2 Material and methods

### 2.1 Ethical considerations

All experimental procedures were approved by the Animal Ethics Committee of the Federal University of Bahia (CEUA/UFBA; protocol no. 20/2022) and conducted in accordance with Brazilian legislation for animal experimentation (Law No. 11.794/2008) and the guidelines of the Conselho Nacional de Controle de Experimentação Animal. The institutional committee operates under national standards aligned with internationally recognized principles of animal welfare. The study was also reported following the ARRIVE 2.0 guidelines.

The experimental design and procedures were conducted in accordance with the principles of the 3Rs (replacement, reduction, and refinement). Throughout the experimental period, animals were monitored daily to assess health status, behavior, and general condition. Handling and sampling procedures were performed by trained personnel to minimize stress and discomfort. Animals had continuous access to water, pasture, and shade. In the event of signs of illness or abnormal behavior, animals received veterinary evaluation and appropriate management. No animals were excluded from the study.

### 2.2 Animals, experimental design, diets, and animal management

Four crossbred (Holstein × Zebu) bulls (375 ± 14 kg BW; 24 ± 1 months of age) fitted with ruminal cannulas (Kehl^®^ 4^′′^ silicone; São Carlos, Brazil) were distributed in a 4 × 4 Latin square design, which was adopted to control animal and period effects, and is widely used in studies with rumen-cannulated animals, allowing repeated measurements and the evaluation of ruminal dynamics under grazing conditions. The animals were maintained on a 1.2 ha pasture of Pangola grass (*Digitaria decumbens*) with supplementation (Table 1).

**Table 1:** Ingredients and chemical composition of the supplement.

Item (g kg ${}^{-1})$	Supplement	Pangola grass
		(*Digitaria decumbens*)
Ground corn	653	–
Soybean meal	230	–
Urea	32.0	–
Mineral^a^	50.0	–
Salt	35.0	–
Composition (g kg ${}^{-1})$ DM		
DM^b^ (g kg^−1^ as fed)	913	335 ± 37.7
OM^c^	854	924 ± 2.00
Ash	146	76.5 ± 2.00
CP^d^	238	98.0 ± 19.0
EE^e^	37.0	47.1 ± 7.00
NDF^f^	105	687 ± 28.5
NFC^g^	463	184 ± 25.2
_i_NDF^h^	36.1	280 ± 39.5
Total digestible nutrients^*i*^	753	611 ± 9.00
Net energy (Mcal kg^−1^ DM)^j^	1.72	1.38
Forage mass (kg ha ${}^{-1})$		
Total forage mass	–	4632 ± 645
Green leaves	–	1262 ± 185
Stem + sheath	–	1220 ± 350
Dead material	–	3080 ± 423
Stocking rate (AU ha ${}^{-1}{)}^{k}$	–	4.96 ± 0.39

^a^ Contained per kilogram of product: 225 g calcium, 160 g phosphorus, 30 g sulfur, 18 g magnesium, 120 mg cobalt, 2500 mg copper, 120 mg iodine, 1800 mg manganese, 36 mg selenium, 5250 mg zinc, 1600 mg fluorine. ^b^ Dry matter. ^c^ Organic matter. ^d^ Crude protein. ^e^ Ether extract. ^f^ Neutral detergent fiber. ^g^ Non-fiber carbohydrates. ^h^ Indigestible neutral detergent fiber. ^i^ According to NASEM (2016) equation. ^j^ According to NASEM (2016) equation. ^k^ Animal unit (standardized to a relative body weight of 450 kg).

The bulls were randomly assigned to the following treatments: (1) control (CON), supplementation of 0.3 % of live weight (BW) without additives; (2) capsaicin (CAP), supplementation of 0.3 % of BW with inclusion of 100 mg kg^−1^ DM of capsaicin (CAPCIN^®^, NutriQuest, São Paulo, Brazil); (3) monensin (MON), supplementation of 0.3 % of BW with inclusion of 20 mg kg^−1^ of total DM of sodium monensin (Poulcox 40^®^, Huvepharma, Bulgaria); (4) capsaicin + monensin (CAPMON), supplementation of 0.3 % of BW with inclusion of sodium monensin 20 mg kg^−1^ DM and 100 mg kg^−1^ total DM of capsaicin.

The dose of capsaicin used in this study was defined based on previous research conducted with grazing beef cattle (Lopes et al., 2025) and further supported by experiments with lactating dairy cows (Vittorazzi Júnior et al., 2022), representing inclusion levels previously evaluated under practical feeding conditions. Likewise, the monensin dose was established according to studies performed with grazing beef cattle, which demonstrated consistent effects on intake, performance, and ruminal fermentation (Coutinho et al., 2023; Guerreiro et al., 2025; Ribeiro et al., 2025).

The animals were maintained under continuous grazing throughout the experimental period and were moved daily at 10:00 (Brasilia time (BRT); UTC−3) to covered feeders installed in the pasture area. The feeders were individualized using movable partitions, forming pens approximately 2 m wide per animal and 4 m in length. Additives were previously weighed and stored in individual containers; at the time of supplementation, the corresponding dose was mixed with the assigned supplement for each animal and offered individually. Supplement intake lasted approximately 40 to 60 min, after which the animals returned directly to the paddock.

The supplementation time was selected to coincide with a period of reduced grazing activity associated with higher ambient temperatures. According to Zanine et al. (2007), between 10:00 and 14:00 (BRT; UTC−3), increased temperatures reduce grazing activity and increase idle time. Thus, providing the supplement during this interval minimizes interference with the main forage intake peaks, which occur primarily in the early morning and late afternoon.

Experimental procedures requiring greater animal restraint and safety were conducted using a handling chute located near the grazing area, followed by immediate release of the animals back to pasture. This approach minimized potential alterations in animal behavior and ensured the maintenance of experimental conditions throughout the study.

### 2.3 Sample collection

*Digitaria decumbens* forage samples were collected from each paddock on 3 different days of each experimental period (1, 6, and 11). Every 3 d, during the rainy season, the average canopy height was measured in order to maintain a height of 30 cm. To analyze forage mass (FM), three grass samples were cut and collected according to the methodology of Barbero et al. (2015).

The collected samples were quantified by weight and divided into two equal portions: half to estimate the total available dry matter of forage (DM, kg DM ha^−1^) of the paddock and the other half to determine the morphological composition of the forage. To analyze the chemical composition of the forage, a grazing simulation was used according to the methodological procedures of Barbero et al. (2015).

### 2.4 Nutrient intake and apparent digestibility of the total tract

The marker technique was used to estimate intake and digestibility, using titanium dioxide (TiO_2_) as an external marker to estimate fecal excretion and indigestible neutral detergent fiber (_i_NDF) as an internal marker to estimate pasture dry matter intake, according to Oliveira et al. (2012) and Detmann et al. (2012), respectively.

Titanium dioxide was inserted directly into the animals' rumen once daily at a dose of 10 g for 10 consecutive days (from day 5 to day 14 of each period). Seven days were intended for the animals' adaptation, and the last 3 d (day 12 to day 14) were for feces collection, directly into the rectal ampulla or after defecation at two different times. The samples were then preserved at - 20 °C to later pool by animal/period, followed by bromatological analyses and the concentration of the markers.

### 2.5 Energy balance and blood metabolites

For energy balance determination, digestible energy (DE) intake was estimated from dietary total digestible nutrients (TDN). Metabolizable energy (ME) was calculated as 0.82 × DE, and net energy (NE) was estimated according to NASEM (2016).

Net energy for maintenance (NEm) was estimated as a function of body weight (BW), adjusted for metabolically active mass. Net energy for gain (NEg) was calculated as the difference between total net energy intake and the energy requirement for maintenance. Empty body weight was assumed to be 81 % of body weight and was determined at the end of each experimental period. Energy balance was obtained by subtracting the energy requirements for maintenance and gain from total net energy intake.

On the 16th day of each experimental period, 10 mL of blood was collected after supplement intake. The timing of sampling was defined to assess the postprandial metabolic response, particularly for metabolites sensitive to recent nutrient intake and ruminal nitrogen dynamics, such as glucose and urea.

After collection, blood samples were centrifuged at 1800 × g for 15 min at room temperature. The serum obtained was transferred to Eppendorf^®^ tubes and stored at - 20 °C until analysis. Serum concentrations of albumin, urea, total protein, glucose, gamma-glutamyltransferase (GGT), aspartate aminotransferase (AST), and cholesterol were determined using colorimetric methods with commercial kits (Doles Reagents, Goiânia, GO, Brazil), and readings were performed using a semi-automatic biochemical analyzer (AJX-1900, Micronal SA, São Paulo, Brazil).

### 2.6 Reticular outflow and nutrient dynamics

Rumen and reticular content sampling were performed between days 16 and 18 of each collection period, every 9 h, strictly following the methodology proposed by Krizsan et al. (2010). After collection, samples were stored at - 20 °C. At the end of each period, samples were thawed and a composite sample was collected for each animal and period, filtered through a 1 mm nylon fabric filter to separate the liquid and solid phases. Each phase was subsequently weighed, pre-dried in a forced-air oven, and ground to 1 and 2 mm particle sizes for subsequent analyses.

Rumen kinetics were determined by removing the entire ruminal content 4.5 h after the morning feeding on day 19 and 2 h before the morning feeding on day 20 of each period (Harvatine and Allen, 2006). During rumen evacuation, 10 % aliquots of the digestive content were separated for precise sampling, filtered, pre-dried (55 °C for 72 h), and ground to 1 and 2 mm particle sizes for subsequent analyses. All procedures, including collection, handling, filtration, and total rumen evacuation, were conducted by trained personnel using careful management techniques to minimize stress and discomfort to the animals, in accordance with current animal welfare regulations and protocols approved by the institutional animal care and use committee. Ruminal metabolism, passage, and digestion rates were determined according to the equations proposed by Oba and Allen (2003).

### 2.7 Laboratory analysis

Samples of pasture, supplement ingredients, feces, ruminal digesta, and reticular digesta were dried at 55 °C for 72 h and then crushed in a 2 and 1 mm sieve. The 2 mm samples were analyzed for the content of indigestible neutral detergent fiber (_i_NDF) according to the procedures of Casali et al. (2009). Potentially digestible NDF (_td_NDF) was calculated by subtracting neutral detergent fiber (NDF) and _i_NDF.

Samples ground to 1 mm of supplement ingredients, pasture and feces were evaluated for their dry matter (DM, procedure 930.15), ash (MM, procedure 942.05), crude protein (CP, procedure 984.13), ether extract (EE, procedure 920.39), and acid detergent fiber (ADF) according to the methods described by AOAC (2000) and neutral detergent fiber (NDF) according to the methodological procedures (Van Soest et al., 1991).

In the samples at 1 mm of the rumen digesta and reticular digesta fractions, DM, MM, and NDF were determined according to the methodologies mentioned above.

### 2.8 Ingestive behavior

Ingestive behavior was assessed on the 10th day of each experimental period, through visual observation over a 24 h period, every 10 min, identifying the animals' activities (feeding (grazing and trough), rumination, or idleness) (Santana Junior et al., 2014). Observations were performed by trained observers using night- and day-vision binoculars, manual counters, and stopwatches. Observers were blinded to the treatments to prevent bias, and inter-observer reliability was evaluated during a training session prior to data collection to ensure consistency in observations.

### 2.9 Rumen fermentation

Between the 16th and 18th day of the period, digesta samples were collected directly from the rumen through the rumen cannula at five different locations (the craniodorsal, cranioventral, ventral, caudoventral, and caudodorsal regions of the rumen). The collection protocol was established to minimize the time the animals were kept away from the pastures, thus reducing the impact on the behavioral cycle. Therefore, the collection times were 0, 3, 6, 9, 12, and 18 h after supplementation, with the first day collections occurring at 10:00 a.m., 02:00 p.m., and 06:00 p.m.; the second day at 12:00 p.m. and 04:00 p.m.; and the third day at 08:00 p.m.

After sampling, the material was filtered and pH was immediately measured using a digital pH meter (ORP 8651, AZ Instrument Corp., Tanzi District, Taichung City, Taiwan). Subsequently, the samples were frozen for volatile fatty acid (VFA) analysis. The analysis of VFA concentrations was carried out according to the methodology proposed by Mathew et al. (1997). The estimation of ruminal methane (CH_4_) production was done using the equations from Moss et al. (2000).

### 2.10 Statistical analysis

The results of intake, total digestibility of nutrients, energy balance, blood metabolites, reticular flow, rumen dynamics, and ingestive behavior were submitted to statistical analysis according to a Latin square design ( 4×4 ) using SAS OnDemand for Academics (SAS Institute Inc., 2026) according to the model below:

1 $$Y_{ijkl}=\mu +a_{i}+p_{j}+d_{k}+\varepsilon_{ijk},$$

where $Y_{ijkl}$ = dependent variable, μ= overall average, $a_{i}$ = random effect of the animal ( i= 1 to 4), $p_{j}$ = random effect of the period ( j= 1 to 4), $d_{k}$ = fixed effect of diet ( k= 4; CON, CAP, MON, and CAPMON), and $\varepsilon_{ijk}=$ random error assumption NID ∼ (0, σ2 ).

The ruminal fermentation variables were analyzed according to the previous design through repeated measures in time, using the PROC MIXED of the SAS, according to the following model:

2 $$Y_{ijklm}=\mu +a_{i}+p_{j}+d_{k}+\varepsilon_{ijk}+t_{m}+t_{m}\times \;d_{k}+\omega_{ijklm},$$

where $Y_{ijklm}$ is the dependent variable value, μ is the overall mean, $a_{i}$ , $p_{j}$ , $d_{k}$ , $t_{m}$ , and $\varepsilon_{ijk}$ were previously described, and $\omega_{ijklm}$ is the random error associated with the time effect assumed NID ∼ (0, σ2 ); $t_{m}$ is the fixed effect of the sampling time ( m= 1 to 7); and $t_{m}$ × $d_{k}$ is the fixed effect of the interaction between diet and time, $d_{k}$ × $t_{m}$ .

To evaluate the differences between the diets, the following orthogonal contrasts were analyzed: C1 = CON vs. CAP + MON + CAPMON; C2 = CAP + MON vs. CAPMON; C3 = CAP vs. MON. All data obtained were submitted to analysis of variance, adopting a significance level of 0.05, and trends were considered when 0.05<P≤0.10 .

Considering the limited number of animals ( n= 4), statistical power analysis was performed for the main response variables using the PROC GLMPOWER procedure of SAS OnDemand for Academics (SAS Institute Inc., 2026). The standard deviation was obtained from the residual variability of the experimental data, considering a total of 16 observations. The results indicated that statistical power was generally low to moderate, which may increase the risk of Type II error.

## 3 Results

### 3.1 Nutrient intake and apparent digestibility of the total tract

Supplementation with CAP and MON increased the total digestibility of OM ( P= 0.050), CP ( P= 0.037), and NDF ( P= 0.048), and tended increase the digestibility of DM ( P= 0.066), EE ( P= 0.088), and TDN ( P= 0.069) compared with CAPMON (contrast 2; Table 2).

**Table 2:** Effect of capsaicin and monensin supplementation on ruminal intake and nutrient digestibility of grazing bulls.

Item	Treatments^a^				SEM^b^	P value^c^		
	CON	CAP	MON	CAPMON		C1	C2	C3
Intake (% BW)								
Dry matter	2.23	2.30	2.41	2.26	0.14	0.997	0.426	0.926
Herbage	1.85	1.91	2.01	1.89	0.13	0.983	0.425	0.859
Intake (kg d^−1^)								
Dry matter	7.11	6.87	7.32	7.07	0.36	0.519	0.640	0.948
Organic matter	6.52	6.30	6.71	6.48	0.32	0.522	0.646	0.948
Crude protein	0.64	0.63	0.66	0.64	0.04	0.486	0.687	0.901
Neutral detergent fiber	4.41	4.24	4.56	4.39	0.12	0.520	0.639	0.951
Ether extract	0.41	0.39	0.42	0.40	0.02	0.490	0.678	0.758
Non-fiber carbohydrates	1.51	1.48	1.54	1.51	0.06	0.547	0.669	0.993
Total digestible nutrients (g kg^−1^)	4.65	4.49	4.39	4.62	0.23	0.520	0.646	0.934
Total tract digestibility (g kg^−1^)								
Dry matter	515	572	655	611	21.3	0.616	0.066	0.091
Organic matter	563	613	694	650	19.2	0.564	0.050	0.085
Crude protein	505	556	631	591	20.6	0.373	0.037	0.053
Neutral detergent fiber	527	604	671	633	20.0	0.873	0.048	0.049
Ether extract	316	447	575	505	41.2	0.826	0.088	0.090
Non-fiber carbohydrates	659	632	748	688	25.4	0.144	0.127	0.597
Total digestible nutrients	581	607	706	655	21.5	0.346	0.069	0.110

^a^ CON = control without addition of additive: CAP = addition of 100 mg of capsaicin (CAPCIN^®^) kg^−1^ of DM; MON = addition of 20 mg kg^−1^ of DM of sodium monensin (Poulcox 40^®^); CAPMON = addition of 100 mg of capsaicin kg^−1^ of DM and 20 mg kg^−1^ of DM of sodium monensin. ^b^ Standard error of the mean. ^c^ Significance level was set at 0.05 and trend considered at 0.10. Diet contrasts C1 = CON vs. CAP + MON + CAPMON; C2 = CAP + MON vs. CAPMON; C3 = CAP vs. MON.

Animals supplemented with MON had higher NDF digestibility ( P= 0.049) and showed a tendency for increased DM digestibility ( P= 0.091), OM ( P= 0.085), CP ( P= 0.053), and EE ( P= 0.090) compared to those supplemented with CAP.

### 3.2 Energy balance and serum blood metabolites, reticulum outflow, and nutrient dynamics

No difference was observed in energy balance and blood metabolism variables (Table 3). The ruminal turnover rate of _td_NDF tended to be higher for animals fed the CON diet ( P= 0.069) (contrast 1; Table 4). A trend was observed in a higher rumen pool of DM for animals supplemented with CAPMON compared with those supplemented with CAP and MON ( P= 0.096). The _td_NDF passage rate ( P= 0.090) and the _td_NDF ruminal turnover rate ( P= 0.094) tended to be higher in animals supplemented with MON than those supplemented with CAP.

**Table 3:** Effect of capsaicin and monensin supplementation on energy balance and serum blood metabolites in bulls.

Item	Treatments^a^				SEM^b^	P value^c^		
	CON	CAP	MON	CAPMON		C1	C2	C3
Intake (Mcal)								
NE	9.54	9.75	10.3	10.0	0.27	0.744	0.409	0.550
ME	15.8	16.2	17.2	16.7	0.45	0.745	0.409	0.546
Production								
NEg (Mcal d^−1^)	6.86	7.09	7.66	7.27	0.25	0.778	0.367	0.585
Energy balance								
NE (Mcal d^−1^)	2.68	2.66	2.68	2.74	0.04	0.757	0.827	0.677
Efficiency (NEg DEI^−1^)	0.35	0.36	0.36	0.36	0.00	0.920	0.391	0.619
Serum blood metabolites								
Albumin (mg dL^−1^)	3.00	3.17	3.17	3.03	0.04	0.394	0.237	0.869
Urea (mg dL^−1^)	17.3	19.1	20.3	22.5	1.33	0.759	0.894	0.171
Total protein (g dL ${}^{-1})$	5.93	6.38	6.40	6.30	0.10	0.510	0.293	0.239
Glucose (mg dL^−1^)	62.6	63.9	66.2	48.9	3.91	0.576	0.255	0.199
GGT (U L^−1^)^d^	22.5	24.0	23.7	24.5	1.18	0.896	0.957	0.643
AST (U L^−1^)^e^	44.6	47.3	43.4	52.6	3.53	0.959	0.505	0.379
Cholesterol (mg dL^−1^)	55.9	59.5	61.2	59.3	1.65	0.877	0.424	0.506

^a^ CON = control without addition of additive: CAP = addition of 100 mg de capsaicin (CAPCIN^®^) kg^−1^ of DM; MON = addition of 20 mg kg^−1^ of DM of sodium monensin (Poulcox 40^®^); CAPMON = addition of 100 mg of capsaicin kg^−1^ of DM and 20 mg kg^−1^ of DM of sodium monensin. ^b^ Standard error of the mean. ^c^ Significance level was set at 0.05 and trend considered at 0.10. Diet contrasts C1 = CON vs. CAP + MON + CAPMON; C2 = CAP + MON vs. CAPMON; C3 = CAP vs. MON. ^d^ Gamma-glutamyltransferase. ^e^ Aspartate aminotransferase.

**Table 4:** Effect of capsaicin and monensin supplementation on rumen dynamics and post-ruminal flow of grazing bulls.

Item	Treatments^a^				SEM^b^	P value^c^		
	CON	CAP	MON	CAPMON		C1	C2	C3
Ruminal volume (L)	62.8	65.0	64.1	65.4	2.54	0.899	0.997	0.763
Ruminal pool (kg of wet matter)	74.9	64.6	73.8	73.9	2.79	0.195	0.933	0.907
Ruminal pool (kg)								
DM^d^	11.1	10.9	11.4	10.8	0.27	0.457	0.096	0.383
NDF^e^	67.1	66.8	68.0	67.8	0.85	0.574	0.723	0.683
_td_NDF^f^	25.3	26.1	24.6	29.0	1.16	0.963	0.472	0.359
Ruminal digestibility (g kg^−1^)								
Relative, DM	654	625	655	611	9.63	0.547	0.410	0.222
Relative, NDF	242	302	319	313	21.1	0.817	0.394	0.216
Relative, _td_NDF	425	552	577	568	34.9	0.722	0.356	0.168
Reticular flow (kg d^−1^)								
DM^d^	3.92	3.97	4.32	4.15	0.17	0.520	0.294	0.479
OM^g^	2.82	2.96	3.09	3.03	0.10	0.915	0.453	0.439
NDF^e^	1.74	1.89	2.02	1.95	0.07	0.957	0.319	0.313
Digestion rate (% h^−1^)								
DM^d^	0.93	1.11	1.10	1.13	0.06	0.710	0.646	0.336
NDF^e^	0.15	0.15	0.16	0.15	0.00	0.624	0.609	0.979
Passage rate (% h^−1^)								
DM^d^	1.51	1.54	1.60	1.62	0.09	0.720	0.754	0.404
NDF^e^	0.11	0.12	0.12	0.11	0.00	0.947	0.440	0.532
_td_NDF^f^	1.08	0.91	1.02	0.79	0.06	0.698	0.534	0.090
Ruminal removal rate (% h ${}^{-1})$								
DM^d^	2.52	2.65	2.70	2.75	0.09	0.962	0.737	0.329
NDF^e^	0.26	0.26	0.28	0.27	0.00	0.819	0.408	0.564
_td_NDF^f^	0.63	0.45	0.64	0.48	0.04	0.069	0.202	0.094

^a^ CON = control without addition of additive: CAP = addition of 100 mg de capsaicin (CAPCIN^®^) kg^−1^ of DM; MON = addition of 20 mg kg^−1^ of DM of sodium monensin (Poulcox 40^®^); CAPMON = addition of 100 mg of capsaicin kg^−1^ of DM and 20 mg kg^−1^ of DM of sodium monensin. ^b^ Standard error of the mean. ^c^ Significance level was set at 0.05 and trend considered at 0.10. Diet contrasts C1 = CON vs. CAP + MON + CAPMON; C2 = CAP + MON vs. CAPMON; C3 = CAP vs. MON. ^d^ Dry matter. ^e^ Neutral detergent fiber. ^f^ Truly digestible neutral detergent fiber. ^g^ Organic matter.

### 3.3 Ingestive behavior

Total feeding time (min d^−1^) ( P= 0.086) tended to be longer when animals were fed the CON diet (contrast 1; Table 5). Animals that received CAPMON supplementation spent more time grazing in min d^−1^ ( P= 0.036) and periods d^−1^ ( P= 0.039), and longer total feeding time (period d ${}^{-1})$ ( P= 0.050). Furthermore, they tended to have a longer total feeding time (min d^−1^) ( P= 0.088) when compared with those fed with CAP and MON supplements (contrast 2; Table 5).

**Table 5:** Effect of capsaicin and monensin supplementation on feeding behavior of grazing bulls.

Item	Treatments^a^				SEM^b^	P value^c^		
	CON	CAP	MON	CAPMON		C1	C2	C3
Activities (min d ${}^{-1})$								
Trough	20.0	22.5	22.5	20.0	0.85	0.343	0.191	1.000
Grazing	530	545	476	525	17.7	0.114	0.036	0.843
Rumination	502	500	525	470	15.3	0.974	0.174	0.311
Idle	388	373	400	425	12.6	0.114	0.752	0.125
TFT (min d^−1^)^d^	550	568	515	545	17.3	0.086	0.088	0.804
TCT (min d^−1^)^e^	1052	1067	1040	1015	12.6	0.114	0.752	0.125
Number of periods d^−1^								
Trough	1.38	1.56	1.56	1.38	0.05	0.343	0.191	1.000
Grazing	36.8	37.8	34.2	36.5	1.20	0.170	0.039	0.884
Rumination	34.9	34.7	36.5	32.6	1.05	0.974	0.174	0.311
Idle	26.9	25.9	27.8	29.5	0.87	0.114	0.752	0.125
TFT (periods d^−1^)^d^	38.2	39.4	35.8	37.8	1.20	0.177	0.050	0.895
TCT (periods d^−1^)^e^	73.1	74.1	72.2	70.5	0.87	0.114	0.752	0.125

^a^ CON = control without addition of additive: CAP = addition of 100 mg de capsaicin (CAPCIN^®^) kg^−1^ of DM; MON = addition of 20 mg kg^−1^ of DM of sodium monensin (Poulcox 40^®^); CAPMON = addition of 100 mg of capsaicin kg^−1^ of DM and 20 mg kg^−1^ of DM of sodium monensin. ^b^ Standard error of the mean. ^c^ Significance level was set at 0.05 and trend considered at 0.10. Diet contrasts C1 = CON vs. CAP + MON + CAPMON; C2 = CAP + MON vs. CAPMON; C3 = CAP vs. MON. ^d^ Total feeding time. ^e^ Total chewing time.

### 3.4 Rumen fermentation

The propionate concentration ( P= 0.056) tended to be lower in the CON diet, while the C2 : C3 ratio ( P= 0.039) was higher in the CON diet (contrast 1; Table 6).

**Table 6:** Effect of capsaicin and monensin supplementation on ruminal fermentation of grazing bulls.

Item	Treatments^a^				SEM^b^	P value^c^					
	CON	CAP	MON	CAPMON		Time	Diet	Diet × Time	C1	C2	C3
pH	6.45	6.43	6.47	6.43	0.02	0.012	0.931	0.997	0.687	0.012	0.998
Total VFA (mmol L^−1^)^d^	46.5	48.8	45.9	45.3	0.46	< 0.001	0.660	0.503	0.793	0.831	0.227
VFA (mmol 100 mmol^−1^)											
Acetate (C2)	74.4	74.3	74.1	74.4	0.46	< 0.001	0.660	0.503	0.793	0.831	0.227
Propionate (C3)	14.5	14.7	14.4	14.7	0.08	0.907	0.014	0.993	0.056	0.012	0.347
Butyrate (C4)	11.1	11.0	11.5	10.9	0.07	0.045	0.858	0.932	0.913	0.405	0.899
C2 : C3 ratio^e^	5.14	5.06	5.17	5.07	0.03	0.959	0.012	0.999	0.039	0.014	0.315
CH_4_ (mmol L^−1^)^f^	15.8	16.5	15.6	15.3	0.16	< 0.001	0.428	0.564	0.496	0.482	0.181

^a^ CON = control without addition of additive: CAP = addition of 100 mg of capsaicin (CAPCIN^®^) kg^−1^ of DM; MON = addition of 20 mg kg^−1^ of DM of sodium monensin (Poulcox 40^®^); CAPMON = addition of 100 mg of capsaicin kg^−1^ of DM and 20 mg kg^−1^ of DM of sodium monensin.^b^ Standard error of the mean. ^c^ Significance level was set at 0.05 and trend considered at 0.10. Diet contrasts C1 = CON vs. CAP + MON + CAPMON; C2 = CAP + MON vs. CAPMON; C3 = CAP vs. MON. ^d^ Volatile fatty acids. ^e^ Acetate : propionate ratio. ^f^ Enteric methane production estimated according to Moss et al. equation (2000): CH_4_ (mmol L^−1^) = 0.45 acetate (mmol L^−1^) - 0.275 propionate (mmol L^−1^) + 0.40 butyrate (mmol L^−1^).

**Figure 1:**
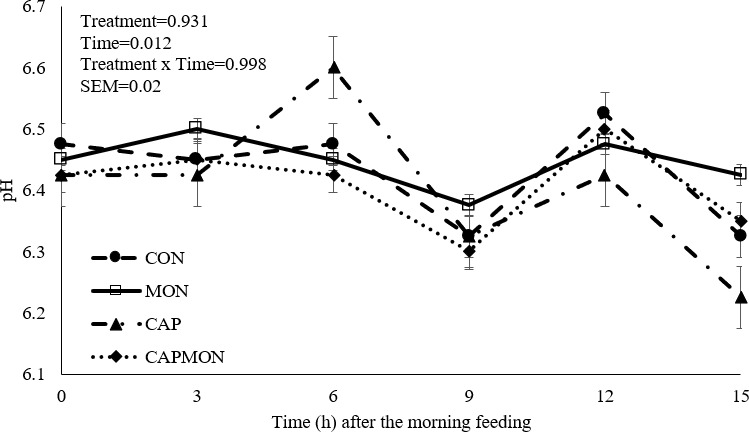
Ruminal pH of grazing bulls supplemented with capsaicin and monensin. CON = control without additive addition; CAP = addition of 100 mg of capsaicin (CAPCIN^®^) kg^−1^ of DM; MON = addition of 20 mg kg^−1^ of DM of sodium monensin (Poulcox 40^®^); CAPMON = addition of 100 mg of capsaicin kg^−1^ of DM and 20 mg kg^−1^ of DM of sodium monensin. SEM: standard error of the mean.

It was found that 6 h after feeding, animals fed the CAP diet had higher pH values compared to the other diets ( P= 0.012, Fig. 1). Animals supplemented with CAP and MON had lower propionate concentration ( P= 0.012) and a higher C2 : C3 ratio ( P= 0.014) when compared with those supplemented with CAPMON (contrast 2; Table 6).

**Figure 2:**
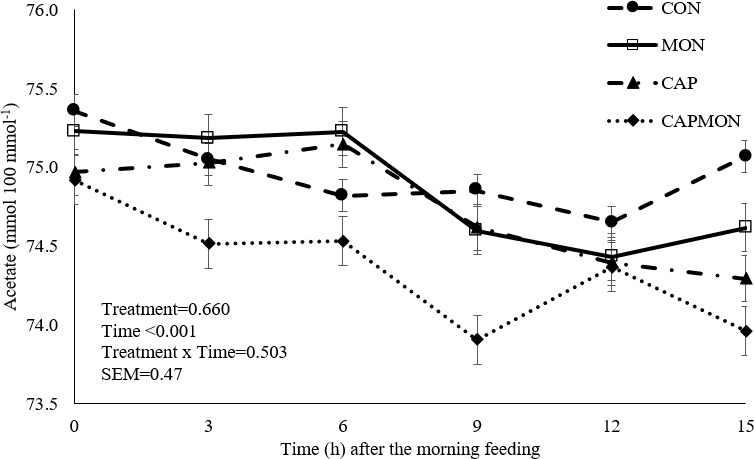
Ruminal acetate concentration of grazing bulls supplemented with capsaicin and monensin. CON = control without additive addition; CAP = addition of 100 mg of capsaicin (CAPCIN^®^) kg^−1^ of DM; MON = addition of 20 mg kg^−1^ of DM of sodium monensin (Poulcox 40^®^); CAPMON = addition of 100 mg of capsaicin kg^−1^ of DM and 20 mg kg^−1^ of DM of sodium monensin. SEM: standard error of the mean.

There was no effect of between diet and time in ruminal fermentation ( P > 0.050). However, an effect of time was observed for pH values ( P= 0.012) (Fig. 1), total VFA ( P<0.001 ), acetate ( P < 0.001) (Fig. 2), butyrate ( P= 0.045), and CH_4_ ( P < 0.001).

## 4 Discussion

Feed additives did not affect dry matter intake (DMI) in grazing bulls maintained under tropical pasture conditions. This response agrees with previous studies conducted in grazing systems, which also reported no changes in DMI with capsaicin or monensin supplementation (Coutinho et al., 2023; Lopes et al., 2025; Miranda et al., 2025). The absence of effects on intake suggests that the inclusion of these additives does not compromise diet palatability or alter the physiological mechanisms associated with the regulation of feed intake. In tropical grazing systems, intake is strongly influenced by factors such as forage availability and quality, canopy structure, and ingestive behavior, which may exert a more pronounced effect than supplementation with additives (Allen et al., 2019). In contrast, inconsistent intake responses, including increases (Orzuna-Orzuna et al., 2024) and decreases (Wood et al., 2016), have been predominantly observed in feedlot systems or in diets with high concentrate inclusion. These differences reinforce the fact that the effects of capsaicin and monensin on intake are highly dependent on the production system, basal diet composition, and additive inclusion rate. In grazing systems, such responses tend to be attenuated due to the greater influence of forage availability, rumen fill, and grazing behavior on intake regulation (Morales et al., 2024).

No overall effect of feed additives on total tract apparent nutrient digestibility was observed when treatments were compared with CON. However, when the individual additives (CAP and MON) were contrasted with their combined use (CAPMON), a reduction in nutrient digestibility was observed, particularly for CP and NDF, in animals receiving CAPMON. These findings indicate that the responses observed in this study were primarily driven by interactions between the additives, suggesting possible overlap or partial antagonism in their mechanisms of action.

Monensin and capsaicin exert their effects primarily through selective modulation of the ruminal microbiota, promoting changes in microbial composition, metabolic activity, and fermentative efficiency. Monensin acts as an ionophore, destabilizing the transmembrane electrochemical gradient of sensitive bacteria, especially Gram-positive species, by impairing ion transport and increasing the energy demand required to maintain intracellular homeostasis, resulting in growth inhibition and, in more severe cases, cell death (Mammi et al., 2021; Moura et al., 2021). This mechanism favors the selection of microbial populations more efficient in converting carbohydrates into propionate, thereby reducing hydrogen and methane production, and improving ruminal energetic efficiency (Schelling, 1984; Mammi et al., 2021).

Complementarily, capsaicin exhibits antimicrobial activity by inducing osmotic stress, compromising membrane integrity, and interfering with gene expression related to bacterial growth, with a greater effect on Gram-positive bacteria (Kurita et al., 2002). In the ruminal environment, this action results in structural changes in the microbial community, including a relative reduction of the genera *Prevotella* and *Roseburia*, and modulation of *Butyrivibrio*, *Faecalibacterium*, *Synergistaceae*, and *Dorea* – microorganisms directly involved in fiber degradation and non-fiber carbohydrate fermentation (Oh et al., 2015). These changes favor a more stable ruminal environment, with increased microbial enzymatic activity, greater efficiency of substrate degradation, and improved digestibility (Orzuna-Orzuna et al., 2024).

However, the combined supplementation of capsaicin and monensin suggests possible overlap or partial antagonism in their mechanisms of action, since both compounds act on similar microbial populations and metabolic pathways related to carbohydrate fermentation and hydrogen metabolism. This similarity may result in redundant or competitive effects when the additives are used simultaneously, thereby limiting additional gains in digestibility (Orzuna-Orzuna et al., 2024).

It is important to emphasize, however, that these mechanisms are inferred and were not directly measured in the present study. Although both monensin and capsaicin exhibit antimicrobial activity, generally against similar bacterial groups, some microorganisms may develop adaptive mechanisms, becoming resistant to monensin, such as *Fibrobacter succinogenes* (Chen and Wolin, 1979). This Gram-negative microorganism is capable of replacing other bacterial populations and maintaining NDF degradation (Schelling, 1984), as well as dry matter degradation in the rumen (Dinius et al., 1976). Thus, the differences observed between additives in digestibility are likely associated with the greater difficulty of ruminal microbiota adaptation to capsaicin.

This hypothesis is further supported by the evaluation of ruminal dynamics, as digestion rate, passage rate, and potentially digestible NDF removal rate tended to be greater in animals supplemented with monensin compared with those receiving capsaicin. These findings support the notion that fibrolytic microorganisms, such as *Fibrobacter succinogenes*, exhibit greater adaptive capacity to monensin than to capsaicin. For crude protein digestibility, the observed effect may be associated with the inhibitory action of capsaicin on proteolytic microbial populations, as reported by Plaizier et al. (2000), Oh et al. (2015), and Betancur-Murillo et al. (2022).

No effect of the additives was observed on animal feeding behavior. However, when contrasting CAP + MON vs. CAPMON, a change in grazing time was detected, mainly associated with capsaicin supplementation, as animals receiving this additive spent more time grazing. Consequently, a tendency for increased total feeding time was observed. Capsaicin activates TRPV1 receptors, which are involved in pain perception and, depending on the dose, may induce discomfort and alter feeding patterns (Frias and Merighi, 2016). Similar changes in feeding behavior were reported by Rodríguez-Prado et al. (2012), who also observed increased feeding time in beef cattle supplemented with capsaicin, attributed to its pungent effect, which may increase meal frequency.

The additives did not affect mean ruminal pH values when compared with the control treatment. Ruminal fermentation parameters reflect microbial metabolic activity, with pH and volatile fatty acid (VFA) concentrations being key indicators of ruminal environmental conditions. When contrasting CAP + MON vs. CAPMON, the isolated use of additives resulted in slightly higher ruminal pH values than their combined use. According to Ricci et al. (2021), the pungent effect of capsaicin may increase salivation rate, thereby enhancing buffer flow into the rumen. In addition, capsaicin may stimulate water intake (Cunha et al., 2020) and modify feeding patterns, leading to the consumption of smaller portions throughout the day without impairing ruminal dry matter intake (Castillo-Lopez et al., 2021), which may contribute to increased pH. However, a meta-analysis conducted by Orzuna-Orzuna et al. (2024) did not identify significant effects of capsaicin on ruminal pH. Similarly, although monensin has been associated with suppression of lactate-producing bacteria (Plaizier et al., 2000), meta-analytical studies indicate no consistent effect of this ionophore on ruminal pH (Moura et al., 2021). Moreover, ruminal pH is governed by a complex interaction among intake, dietary fermentation, production and absorption of VFAs, ammonia production, and salivary secretion (Fandiño et al., 2020), and the differences observed in the present study were small and of limited biological relevance.

Animals supplemented with additives tended to present higher ruminal propionate concentrations, reinforcing the fact that phytogenic additives such as capsaicin may exert ruminal effects similar to conventional rumen modifiers, such as monensin. When these compounds promote selective inhibition of Gram-positive bacteria, there is a shift toward Gram-negative populations, resulting in decreased acetate concentrations and increased propionate concentrations in the rumen (Oh et al., 2018). When contrasting CAP + MON vs. CAPMON, combined supplementation resulted in greater propionate production and a lower acetate : propionate ratio (C2 : C3). Considering that CAPMON provided the same individual concentrations of each additive, this strategy resulted in greater total additive supply (g d^−1^), enhancing microbial selection and more strongly favoring the development of propionate-producing populations.

## 5 Conclusions

The use of capsaicin and/or monensin at the tested doses can be applied in animal production systems. The association of capsaicin with monensin was effective in increasing the molar proportion of ruminal propionate, indicating a potential improvement in ruminal fermentation patterns. Further research is needed to elucidate the mechanisms of action underlying the combined use of these additives.

## Data Availability

The data that support this study will be shared upon reasonable request to the corresponding author.
